# The Art of War: Beyond Memory-one Strategies in Population Games

**DOI:** 10.1371/journal.pone.0120625

**Published:** 2015-03-24

**Authors:** Christopher Lee, Marc Harper, Dashiell Fryer

**Affiliations:** 1 Institute for Genomics and Proteomics, University of California, Los Angeles, CA, USA; 2 Dept. of Chemistry & Biochemistry, University of California, Los Angeles, CA, USA; 3 Dept. of Computer Science, University of California, Los Angeles, CA, USA; 4 Molecular Biology Institute, University of California, Los Angeles, CA, USA; 5 Department of Mathematics, Pomona College; Tianjin University of Technology, CHINA

## Abstract

We show that the history of play in a population game contains exploitable information that can be successfully used by sophisticated strategies to defeat memory-one opponents, including zero determinant strategies. The history allows a player to label opponents by their strategies, enabling a player to determine the population distribution and to act differentially based on the opponent’s strategy in each pairwise interaction. For the Prisoner’s Dilemma, these advantages lead to the natural formation of cooperative coalitions among similarly behaving players and eventually to unilateral defection against opposing player types. We show analytically and empirically that optimal play in population games depends strongly on the population distribution. For example, the optimal strategy for a minority player type against a resident TFT population is ALLC, while for a majority player type the optimal strategy versus TFT players is ALLD. Such behaviors are not accessible to memory-one strategies. Drawing inspiration from Sun Tzu’s the Art of War, we implemented a non-memory-one strategy for population games based on techniques from machine learning and statistical inference that can exploit the history of play in this manner. Via simulation we find that this strategy is essentially uninvadable and can successfully invade (significantly more likely than a neutral mutant) essentially all known memory-one strategies for the Prisoner’s Dilemma, including ALLC (always cooperate), ALLD (always defect), tit-for-tat (TFT), win-stay-lose-shift (WSLS), and zero determinant (ZD) strategies, including extortionate and generous strategies.

## Introduction

The Prisoner’s Dilemma (PD) [1] is a two player game with a long history of study in evolutionary game theory [2] and finite populations [3]. Work on time-averaged fitness [4] and interaction neighborhood size on regular lattices [5], is of particular interest. Payoffs for the Prisoner’s Dilemma are usually defined via a game matrix $\left(\begin{array}{cc} R & S\\ T & P \end{array}\right)$ with *T* > *R* > *P* > *S* and often 2*R* > *T* + *S*. A special case known as the *donation game* is given by *T* = *b*, *R* = *b* − *c*, *P* = 0, *S* = −*c*, with 0 < *c* < *b*. There are many well-known strategies for the Prisoner’s Dilemma, such as ALLC (always cooperate), ALLD (always defect), tit-for-tat (TFT) [6] and win-stay-lose-shift (WSLS) [7]. The discovery of zero determinant strategies by Press and Dyson [8] has invigorated the study of the Prisoner’s Dilemma, including the evolutionary stability of these strategies in population games and their relationship to and impact on the evolution of cooperation [2] [9] [10] [11] [12] [13] [14]. In a tournament emulating the influential contest conducted by Axelrod [15], Stewart and Plotkin show that some zero determinant (ZD) strategies are very successful; Adami and Hintze [13] have shown that ZD strategies are evolutionarily unstable in general, but can be effective if opponents can be identified and play can depend on the opponent’s type (including versus itself). In particular, how a strategy fares against itself becomes crucial in population games.

Many strategies for the Prisoner’s Dilemma have been studied in a huge array of contexts, and it is often found that simpler strategies can beat more complex strategies (e.g. TFT won early repeated Prisoner’s Dilemma tournaments [15]). Commonly PD strategies are formulated as first-order Markov processes known as memory-one strategies, i.e. strategies in which the next move depends only on the last game outcome. Such a process is described by a *strategy vector* of four probabilities denoting the probability that the player will select to cooperate (C) based on the previous round of play: (*Pr*(*C*∣*CC*), *Pr*(*C*∣*CD*), *Pr*(*C*∣*DC*), *Pr*(*C*∣*DD*)). Press and Dyson suggested that some memory-one strategies can dominate more complex strategies; specifically, that using higher-order history does not help versus a ZD strategy [8] in head-to-head interactions. Stewart and Plotkin have also argued that a generous ZD strategy can be robust against *any* invading strategy (i.e. no invader can achieve better than neutral fixation probability) [9] under a set of assumptions including weak selection. (We will refer to these robust strategies as ZDR, and extortionate ZD strategies as ZD_*χ*_; see Methods for details). In population games, Adami and Hintze indicated that *tag* information identifying which players are of the opposing type can significantly increase evolutionary success [13]. They also suggested that it is possible to recognize an opponent’s strategy from the history of play. Can information from past history, ignored by memory-one strategies, improve evolutionary success?

**Fig 1:**
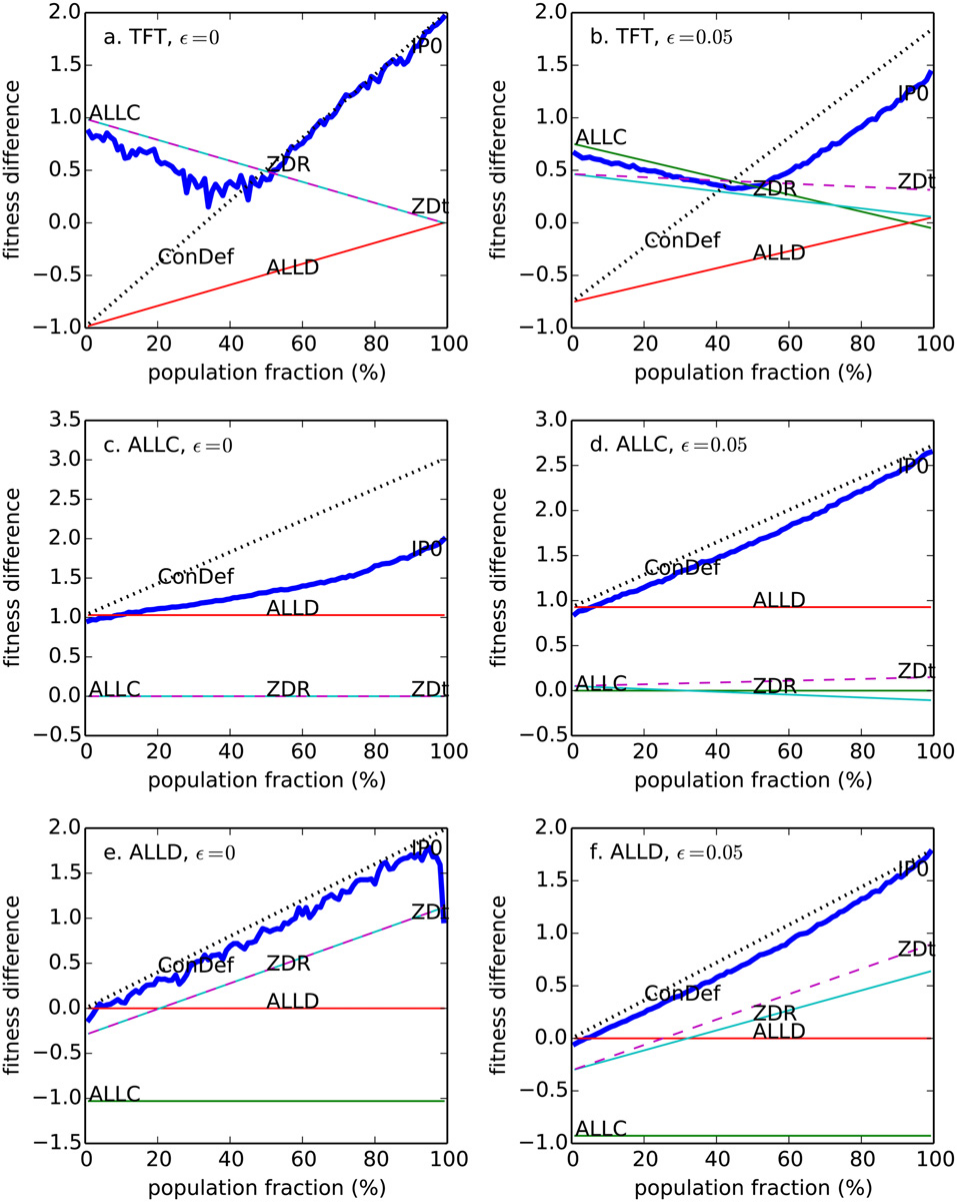
Mean fitness difference $\bar{S_{I}}-\bar{S_{G}}$ as a function of the invader’s population fraction. Plotted for several different invading strategies (I = ALLC, ALLD, ZDR, ConDef, ZD_t_) vs. several different resident strategies: TFT (A, B); ALLC (C, D); ALLD (E, F).

A player capable of utilizing the history of play has the following potential advantages:

- *self-recognition*: as Adami and Hintze pointed out, a player type that is capable of recognizing other players using instances of its strategy can gain an advantage by always cooperating with other instances but playing a quite different strategy versus other types.
- *frequency-dependent strategy optimization*: a player can use its history of play against all its opponents to estimate *what fraction of the population* is composed of its own type. We will show that the best strategy against a given opponent type can change dramatically depending on the population proportion of its type (which we will denote as *f*). For example, when in the minority (*f* ≈ 0) it is optimal to cooperate vs. a resident TFT population, but when in the majority (*f* ≈ 1) it becomes optimal to defect vs. TFT players (see Fig. 1).

In this paper we assess the value of these two approaches both analytically and via simulations versus a variety of traditionally successful strategies and ZD strategies; but our results are not limited to such opponents, nor for that matter to the Prisoner’s Dilemma game. We first present analytic results on the average fitness benefit of frequency dependent strategy optimization versus a range of well-known memory-one Prisoner’s Dilemma strategies. We then use simulations to assess the practicality of implementing these two approaches purely from the observed history of play. In other words, for a player to perform self-recognition and estimate its population fraction *f* solely from its game outcomes versus its opponents, with no tag information provided, and even with significant levels of noise (i.e. players’ moves are flipped with error probability *ε*). We refer to our implementation of frequency-dependent strategy optimization as an *information player* IP_0_. We empirically test IP_0_’s self-recognition accuracy under noise and measure empirical fixation probabilities for IP_0_ invading well-known memory-one strategies and for a resident IP_0_ population being invaded by these memory-one strategies. In general we find that IP_0_ is more robust than memory-one strategies, in that it is uninvadable by memory-one strategies, while achieving near-maximum invasion success (among fixation probabilities of the other opponent strategies) against each memory-one opponent.

## Results

We begin by analyzing whether there is any theoretical advantage to switching strategies at different values of the population fraction *f* against a given memory-one opponent type. We start with the “best case scenario” provided by the *tag assumption*, in which a player knows which players are of the same type (instances of the same strategy), and hence can both play differently based on opponent type in each pairwise interaction as well as determine the population fraction *f* of its type accurately.

### Stationary Score Analysis of Frequency-dependent Strategy Optimization

The long run evolutionary fitness of a player of invading type *I* is determined by its mean stationary score relative to that of players of the opposing group *G*. Let the population consist of *m* players of type *I* in the population and *N* − *m* of type *G*. Let $\bar{S_{II}},\bar{S_{IG}},\bar{S_{GI}},\bar{S_{GG}}$ be the average stationary scores of players in pairwise interactions of the two types. Then the difference in mean stationary payout is given by

$$\begin{array}{c} \bar{S_{I}}-\bar{S_{G}}=\frac{m-1}{N-1}\bar{S_{II}}+\frac{N-m}{N-1}\bar{S_{IG}}-\frac{m}{N-1}\bar{S_{GI}}-\frac{N-m-1}{N-1}\bar{S_{GG}} \end{array}$$ (1)

For large populations, this simplifies to

$$\begin{array}{c} \bar{S_{I}}-\bar{S_{G}}\to f\left(\bar{S_{II}}-\bar{S_{GI}}\right)+\left(1-f\right)\left(\bar{S_{IG}}-\bar{S_{GG}}\right)\;\text{as}\;N\to \infty \end{array}$$

An optimal strategy for player *I* is simply one that maximizes $\bar{S_{I}}-\bar{S_{G}}$. Note that this is strongly dependent on the population fraction *f* = *m*/*N*; for small *f* (*m* ≪ *N*), $\bar{S_{I}}-\bar{S_{G}}$ is dominated by the $\bar{S_{IG}},\bar{S_{GG}}$ terms; whereas for large *f* it is dominated by the $\bar{S_{II}},\bar{S_{GI}}$ terms. The two-player game considered by Press & Dyson is a special case of this spectrum; specifically, *N* = 2 is the only case where there is only one possible mixture value of *m* (hence no possibility of frequency-dependent strategy optimization), and the difference in mean stationary payout reduces to $\bar{S_{I}}-\bar{S_{G}}=\bar{S_{IG}}-\bar{S_{GI}}$.

**Fig 2:**
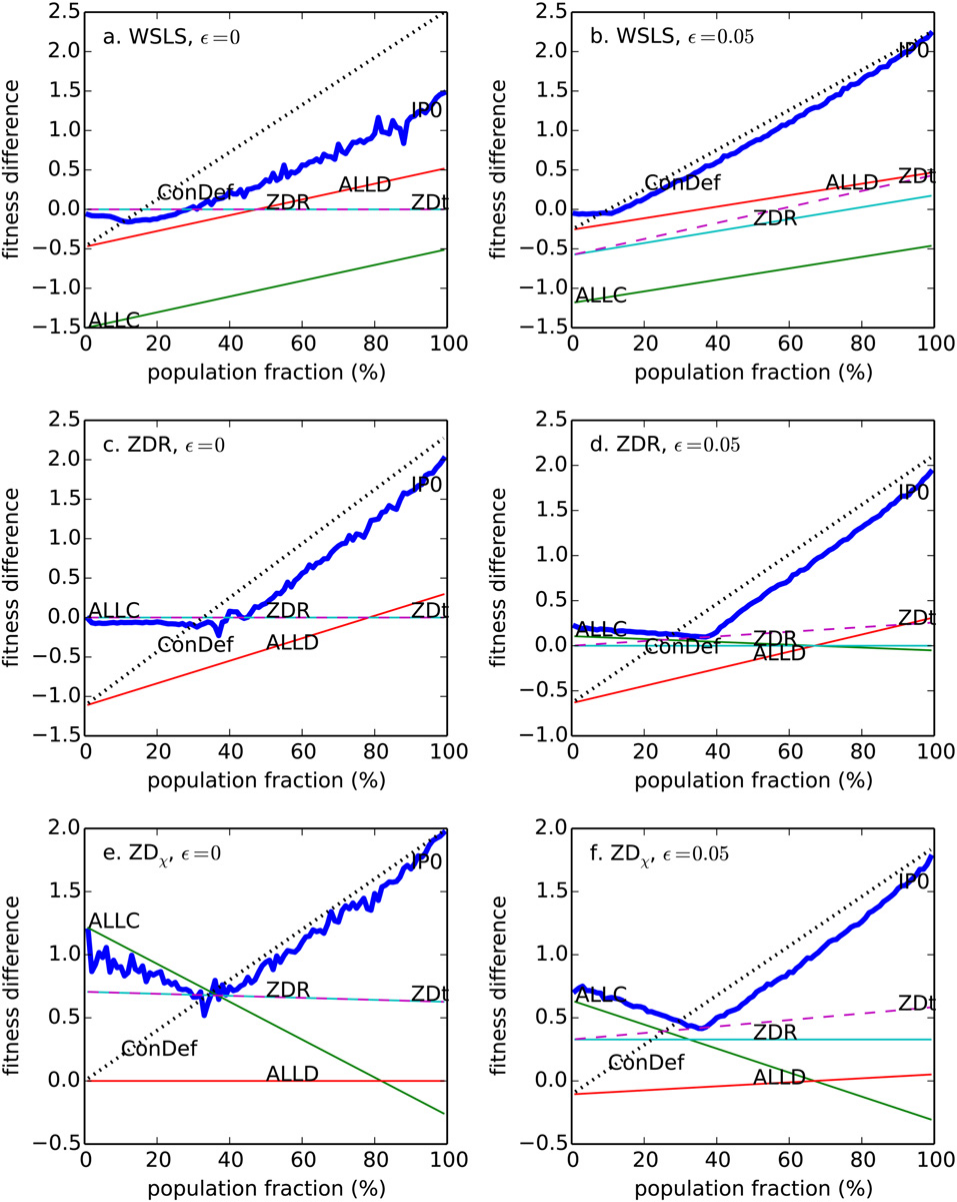
Mean fitness difference $\bar{S_{I}}-\bar{S_{G}}$ as a function of the invader’s population fraction. Plotted for several different invading strategies (I = ALLC, ALLD,ZDR, ConDef, ZD_t_) vs. several different resident strategies: WSLS (A, B); ZDR (C, D); ZD_*χ*_ (E, F).

Fig. 1 and Fig. 2 show $\bar{S_{I}}-\bar{S_{G}}$ as a function of population fraction *f*, for a variety of established strategies, computed from their long-term (stationary) scores [8]. Several basic conclusions emerge from these plots. First, no strategy is universally optimal against all opponents. For example, at low population fractions, ZDR is optimal against WSLS, whereas ALLC is optimal against ZD_*χ*_. Second, even against a single opponent, typically no strategy is optimal at all population fractions. For example, against WSLS, ZDR scores better than ALLD at low population fractions, but worse than ALLD at high population fractions.

Even at a single given point on such a score plot, it is commonly not optimal for players of type *I* to play the same strategy vector with each other as with the opposing players of type *G*. For example, at high population fractions, playing ALLD vs. the opponent (ensuring $\bar{S_{GI}}\leq P$) while playing ALLC with each other (yielding $\bar{S_{II}}=R$) maximizes $\bar{S_{I}}-\bar{S_{G}}\to R-P$. Hintze and Adami have posited a theoretical strategy, *Conditional Defector* (ConDef), able to play different strategies depending on the opponent type. Assuming that ConDef is given the correct *tag* for the type of each player, ConDef cooperates with other ConDef players and defects versus players of the opposing type [13]. (They also defined a tag-based ZD player ZD_t_ that cooperates with other ZD_t_ players and plays a ZD strategy against the opposing type). Lastly, it is striking that even traditionally successful strategies such as WSLS and ZDR are vulnerable to invasion, because at low population fractions an invader can achieve parity (neutral selection) vs. these strategies, while at high population fraction IP_0_ can gain a crushing advantage over them (by switching to what is essentially ConDef).

Taken together, these results suggest that information gleaned from the history of previous game outcomes can yield several basic advantages for choosing moves in the subsequent rounds, all of which are crucial for maximizing $\bar{S_{I}}-\bar{S_{G}}$:

- Player *I* can generate type tags from the history of play, i.e. infer which individual players are instances of its own strategy and which are of different strategies (i.e. of type *G*; we refer to this as *identification*)
- Player *I* can estimate player *G*’s strategy vector, enabling it to choose the optimal counter-strategy;
- Player *I* can estimate what fraction of the population consists of players of type *G*.

### Test Implementation of an Information Player

These calculations suggest that information from a player’s complete history of game outcomes could in theory improve its evolutionary fitness in a poulation game (for example by enabling it to choose the optimal strategy for its current population fraction). However, they do not tell us whether this would actually be feasible or useful in practice. To assess this, we sought to implement a basic player algorithm that infers type identification, population fraction, and optimal strategy from a player’s observed game outcomes. We refer to a player that uses such information (history or type tags) as an *information player* (IP).

It may be helpful to understand our approach as a recapitulation of long-standing principles of competitive strategy, as summarized in Sun Tzu’s *The Art of War*:

*The general who wins the battle makes many calculations in his temple before the battle is fought. The general who loses makes but few calculations beforehand.*

*Know your enemy and know yourself, find naught in fear for 100 battles.*

*…what is of supreme importance in war is to attack the enemy’s strategy.*

*One defends when his strength is inadequate, he attacks when it is abundant.*

*– Sun Tzu, The Art of War*

Our information player implementation IP_0_ embodies these principles as follows:

- *Know your enemy*. Rather than seeking to maximize its *score*, IP_0_ initially seeks to maximize its *information* about another player’s strategy vector. For the first 10 rounds vs. a specific player, IP_0_ selects its plays, either cooperate (C) or defect (D), solely to maximize its information yield about the other player’s strategy vector probabilities. We refer to this as the *information gain phase*. The four probabilities of the opponent’s strategy vector are estimated from these rounds of play and are continually refined in subsequent rounds.
- *Know yourself*. Each IP_0_ individual attempts to identify whether each other player is also IP_0_, based purely on whether it appears to “play like me” (choose the same moves an IP_0_ would have chosen). In particular, the information gain phase produces a unique pattern of play, that can be quickly recognized (within 3—10 moves), even in the presence of random noise (randomly flipped moves). In this sense, the information gain phase may be considered analogous to the handshake phase that has been observed in evolution of finite state automata [16].
- *Attack the enemy’s strategy*. In subsequent rounds, each IP_0_ seeks to maximize its own average score (and by extension that of all IPs in the population) vs. that of the opposing player type. Specifically, it always seeks to cooperate with other IP_0_ individuals; versus the opposing type, it chooses the optimal strategy vector based on its estimate of the opposing type’s strategy vector. As rounds proceed, each IP_0_ continues to update its estimate of opponents probabilities, and adjusts its play as needed to maximize its average score difference.
- *One defends… one attacks…* IP_0_ naturally switches effective strategy depending on the proportion of IP_0_ in the population, and the opponent strategy. Commonly, IP_0_ initially cooperates with the opposing type, when IP_0_ is in the minority, and later defects against the opposing type, when IP_0_ is in the majority. Note that an IP_0_ player uses all of its information (all of its histories in the current population game) to make this decision, and when it switches strategy, it does so simultaneously against all players it considers not IP_0_.

Our example implementation (designated IP_0_) uses machine learning techniques to perform frequency-dependent strategy optimization from a player’s observed game outcomes (see Methods for details). It should be emphasized that each IP_0_ player in a population acts completely independently; different IP_0_ in a population share no information and do not communicate. Note also that when a new IP_0_ player is born, it starts with zero information about other players (no history data), and inherits no information from its parent.

### Accuracy of Identification and Robustness to Noise

That identification of opponent strategies is useful as shown in Fig. 1 and Fig. 2 highlights a crucial question: in the absence of strategy-indicating labels, can an information player determine the identity of other players (*I* vs. *G*) rapidly and accurately from the history of play?

When encountering a new opponent (of unknown strategy), IP_0_ begins with an *information gain* phase (infogain, see Methods). This phase seeks to collect maximal information about the opponent’s strategy vector, and at the same time estimates the likelihood that the opponent is also an IP_0_ player; specifically whether the opponent is also playing by the infogain rule. Thus the infogain phase achieves self-recognition by a most basic principle, “does the opponent play the way I would?” (i.e. chooses the same moves as IP_0_).

**Fig 3:**
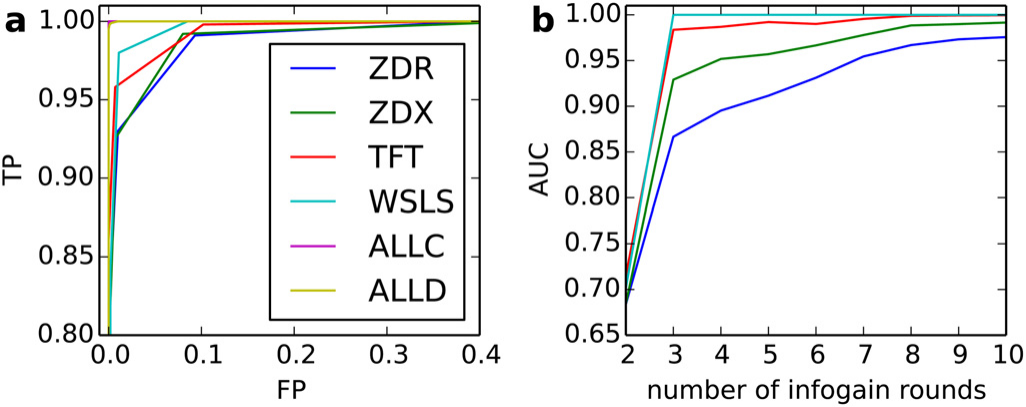
Accuracy of information gain phase. A: ROC for *ε* = 0.05 and 10 infogain rounds. Vertical axis: true positives, Horizontal axis: false positives. ZDR is the hardest strategy to recognize among those tested. B: AUC for IP against ZDR for *ε* = 0, 0.01, 0.05, 0.1.

This approach can rapidly discriminate non-IP_0_ players. In the absence of random noise (move errors), it is simple: the very first move that does not match the expected infogain move exposes the opponent as non-IP_0_, and this typically occurs within the first 3 moves. To make identification more challenging, we assessed the effect of random noise, by randomly flipping each player’s move with probability *ε*. Now IP_0_ must assess observed mismatches probabilistically, e.g. by computing the probability that the observed mismatches could have arisen from another IP_0_ player due solely to random noise (see Methods). This can achieve good discrimination, at the cost of a few extra rounds. Fig. 3A shows Receiver-Operator Characteristic (ROC) curves for discrimination of non-IP_0_ players (vertical axis, True Positives) vs. IP_0_ players (horizontal axis, False Positives) after 10 rounds of play under 5% noise. Corner strategies such as ALLC and ALLD were identified perfectly (i.e. 100% TP at 0% FP), while the most difficult case (ZDR) was identified with 98% accuracy at a false positive rate of only 10%. Concretely, for *N* = 100 players, a single IP_0_ player invading a resident ZDR population could confidently identify 97 of the 99 ZDR players, while having a 90% probability of recognizing any new IP_0_ player within just 10 rounds of play.

To summarize the speed of this process and its sensitivity to noise, we computed a standard measure of discrimination accuracy (AUC, Area Under the Curve, the integral of the ROC curve) for the hardest case (ZDR), and plotted it as a function of number of infogain rounds and for different levels of noise (Fig. 3B). At zero noise, perfect discrimination (AUC = 1) was achieved after just 3 rounds; with up to 10% noise, AUC accuracies of 87–98% were attained after just 3 rounds. Even at 10% noise, AUC accuracy of greater than 97% was attained after 10 rounds.

### Empirical Fixation Probabilities for IP_0_ vs. Memory-one Strategies in Evolutionary Simulations

To assess whether IP_0_ can invade other strategies and resist invasion, we conducted a large number of simulations of IP_0_ versus other strategies for the Prisoner’s Dilemma (Table 1). Such simulation studies are necessary because the performance of IP_0_ will depend on details of its specific implementation in actual play, which are not accessible to closed-form analytic solutions [17]. Our simulations follow a combination of the previous protocols of Adami and Hintze [13] and Stewart and Plotkin [9] (see Methods for details):

- We simulate a well-mixed population of *N* = 100, typically beginning with a single player of the invading type (*m* = 1) within a resident population of the opposing type, and continuing until one type goes extinct.
- No tag (type) information about any player is supplied.
- Every generation, each player plays one move versus each other player. In the case of memory-one strategies, this move is conditioned on its last game outcome versus that player (i.e. in the previous generation).
- Moves are randomly flipped with probability *ε* (typically *ε* = 0.05).
- Payoffs for each game outcome are drawn from the donation game matrix, and the fitness of each player is simply the average of all its payoffs in that generation.
- Each generation, one player dies / is born according to the exponential imitation dynamic (with selection strength *σ* = 1).
- Games involving a new player (born in the previous generation) have no last game outcome, so in this case players apply their standard first move mechanism.

**Table 1:** Fixation odds ratios *ρ*/*ρ*_*neutral*_ of a single row player invading a population of *N* − 1 = 99 column players.

	IP_0_	ALLC	ALLD	TFT	WSLS	ZDR	ZD_*χ*_
IP_0_		58.10	5.50	43.60	1.96	16.30	51.01
ALLC	0		0	49.48	0	21.14	54.78
ALLD	0	59.38		0	0.05	0	0
TFT	0	0	3.68		0	0	9.74
WSLS	0	34.72	0	7.11		0.32	21.16
ZDR	0	0	0.86	24.07	0		27.55
ZD_*χ*_	0	0	1.61	0	0	0	

At least 10,000 simulations were performed for each pair of types, with an ambient error rate of *ε* = 0.05. For IP_0_, p-values for the null hypothesis of neutral fixation is *p* = 5 × 10^−10^ for ALLD and *p* < 10^−26^ otherwise.

Table 1 lists the fixation odds ratio of each strategy versus each other strategy, determined empirically via simulation (specifically, it gives the ratio *ρ*/*ρ*_*neutral*_, where *ρ* is the observed fixation probability, and *ρ*_*neutral*_ = 1/*N* is the fixation probability expected under neutral selection, so a table value of 1.0 indicates neutral selection). In no case was IP_0_ successfully invaded by any other strategy. By contrast, IP_0_ is able to invade all other strategies, with a fixation probability greater than a neutral mutant (*ρ* > *ρ*_*neutral*_), and in all cases is either the best or second best invader (i.e. largest or second largest value in each column). In the language of the Moran process, IP_0_ has higher relative fitness versus all other strategies, and as a resident strategy is evolutionarily robust (defined as *ρ* ≤ *ρ*_*neutral*_ for all invaders [18]) tested. Qualitatively similar results hold for other population sizes *N* ≈ 30 or greater. We also simulated with a Moran selection rule, where each round one player is selected to reproduce proportionally to fitness and one player is selected to be replaced uniformly at random [19] [3]. Results were similar, as are results for simulations using the standard Prisoner’s Dilemma score matrix (as in [8], instead of the donation game matrix). In principle, IP_0_ should excel in any asymmetric game with similar updating rules (it is not designed specifically for a particular game or updating rule).

These values reveal much about how IP_0_ competes against other players. IP_0_ is nearly as effective against ALLC as ALLD is, and quickly learns to exploit ALLC, but has a slightly smaller fixation probability because of the info gain phase. IP_0_ also fares well against ALLD, behaving much like TFT in that it cooperates with other (identified) IP_0_ individuals and defects against ALLD. Outcomes versus ALLD are sensitive to initial population proportion. An invading subpopulation of 10 IP_0_ has an empirically computed fixation probability of *ρ* ≈ 0.5 (versus a neutral fixation probability of *ρ* = 0.10).

Versus TFT, IP_0_ does not fall prey to the mismatches due to errors that TFT is prone to [3], but may suffer versus TFT in the infogain phase, and so does not fare quite as well as ALLC, but has a higher chance to invade than all other players. Among all strategies in our simulations, IP_0_ is the only strategy to have a fixation probability greater than a neutral mutant (*ρ*_*neutral*_ = 1/*N*) versus all other strategies, and the only strategy resistant to invasion by all other strategies.

In general, the ability of IP_0_ to invade other strategies appears to correlate with its fitness difference vs. those strategies at low population fractions (i.e. *m* ≈ 1, see Figs. 1–2). For those where IP_0_ can immediately achieve a strongly positive stationary score difference (e.g. vs. ALLC, TFT, ZDR, ZD_*χ*_), it can invade with high fixation probabilities. For those where IP_0_ is confined to neutral score for low values of *m* (e.g. vs. ALLD, WSLS), its fixation probabilities are lower.

Regarding the effect of ambient noise, smaller values of *ε* make TFT more challenging to infiltrate, however at *ε* = 0.01 the fixation probability of an IP_0_ mutant is still 8 times the neutral probability. This dependence is due to the relatively large number of rounds needed for TFT to reach its stationary distribution versus some other strategies, and this prolongs the time needed to invade an ambient population of TFT players. IP_0_ is apparently uninvadable by TFT at *ε* = 0.01 and *N* = 100, but was invaded once in 10017 simulations for *N* = 40.

### Robust Zero Determinant Strategies

**Fig 4:**
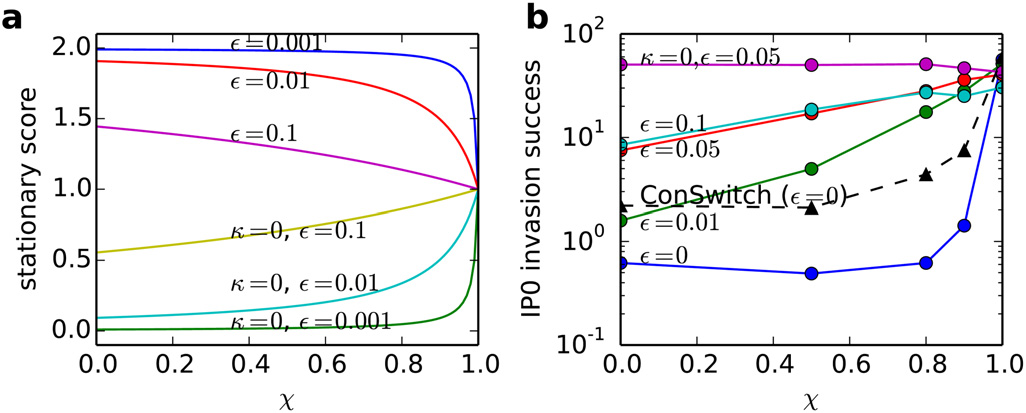
Performance analysis of IP_0_ vs. ZD strategies. A: Stationary self-score $\bar{S_{GG}}$ of ZDR and ZD_*χ*_ (*κ* = 0) at different levels of noise *ε*. B: Invasion success of IP versus ZD strategies (log-scale, fixation probability of an IP_0_ invader, normalized so 1.0 = neutral selection) for different levels of noise *ε*. The top plot is extortionate (*κ* = 0) while the lower three plots have *κ* = *B* − *C* so the ZD strategies are cooperative [20]. As the value of *χ* increases, the fixation probability of IP increases. As the amount of noise decreases, the fixation of our implementation of IP approaches the neutral fixation. With no noise, an optimal IP player (ConSwitch, see text) can empirically invade ZDR at twice the neutral probability (20 out of 1000 simulations with the information phase replaced with tags).

Stewart and Plotkin have outlined a series of assumptions under which ZDR strategies are robust to all other IPD strategies [9]. One implicit assumption in this argument is that player types cannot be identified, either by a tag as described by Adami and Hintze [13] or by statistical inference from the history of play as performed by IP_0_. As shown in Fig. 2C, ZDR strategies are vulnerable to invasion by such information players, because the ZDR can at best guarantee neutral selection i.e. ($\bar{S_{I}}-\bar{S_{G}}=0$) vs. the IP invader at low population fractions (*m* ≈ 1), whereas when the IP invader is in the majority it can gain a strong selective advantage ($\bar{S_{I}}-\bar{S_{G}}⪢0$). In simulations, we found that a tag-based IP (ConSwitch) invades ZDR at much higher than neutral fixation probability (*ρ*/*ρ*_*neutral*_ ≈ 1.6, see Fig. 4), and that IP_0_ achieved better than neutral invasion success against ZDR for *χ* ≥ 0.8 even at zero noise (*ε* = 0). We wish to emphasize that our IP_0_ implementation clearly falls far short of the theoretical IP performance limit as indicated by ConSwitch. This mirrors what we saw at low population fractions in Fig. 2B, where IP_0_ falls short of the perfect (neutral) score that ConSwitch attains vs ZDR. This shortfall is due to the cost of the infogain phase in the current IP implementation, which indicates a clear direction for improvement of our IP implementation.

A second factor that renders ZDR easily prone to invasion by IP_0_ is the effect of noise. Even low levels of noise (e.g. *ε* = 0.01) enabled IP_0_ to invade ZDR at better than neutral fixation probability at all values of *χ* (Fig. 4). In general, noise appears to degrade the performance of Markov players such as ZDR even more than it degrades the performance of IP_0_. Specifically, noise reduces ZDR’s ability to cooperate with itself (i.e. the fraction of ZDR-ZDR game outcomes that are CC), and hence its stationary score (see Fig. 4), more than it reduces IP_0_’s ability to cooperate with itself (because its self-recognition algorithm is robust to noise, and its self-strategy—ALLC—is less affected by noise than ZDR is).

## Discussion

Fixation probabilities for zero-determinant strategies were studied by Stewart and Plotkin [20] in the case of weak selection. For weak selection, no history, zero noise, and stationary payouts, the robustness results of Stewart and Plotkin are not contradicted by our empirical results (likewise for the strong selection results in [21]). Our results, however, indicated that with tagging of player strategies, either given or inferred from the history of play, robust zero determinant strategies can be invaded for non-weak selection and noise. This should not be surprising from Fig. 1 and Fig. 2. ZDR is not generally able to invade IP_0_ nor the variety of strategies that IP_0_ is able to invade. For instance, ZDR is neutral versus e.g. ALLC (with *ε* = 0), whereas IP_0_ can invade ALLC easily at the same level of noise. Note that whereas IP_0_ is always able to invade ZDR strategies, even at zero noise, neither ZDR strategies nor any of the other strategies tested is ever able to invade IP_0_.

Our results indicate IP_0_ is robust to invasion against all the opponents in Table 1. That this generally holds is simply a consequence of the fact that IP_0_ maximizes the mean score difference with its opponents while obtaining the cooperative payout when playing itself. Therefore, once the information gain phase is over, IP_0_ will fixate at least as well as a neutral mutant strategy, and typically much more often. For IP_0_ to be invaded or resisted better than a neutral mutant, the opponent strategy must somehow exploit the manner in which IP attempts to gain information (perhaps by mimicking IP_0_ to be misidentified as another IP_0_ player), or the information gain phase must be too costly (for instance in a very small population). We conjecture that for sufficiently large populations IP_0_ is robust to invasion against all memory-one strategies, and also that IP_0_ is neutral or better as an invader of memory-one strategies (with exceptions occurring mainly for small ambient noise and/or weak selection).

While we have discussed our results in the context of the Prisoner’s Dilemma, IP_0_ is effective in principle in *all* population games without significant modification. For any game matrix, IP_0_ will still identify other players’ strategies and maximize the difference in stationary payout. Information players should fare well in a variety of other contexts, including asymmetric games and population games on graphs, time-averaged fitness [4], and increased interaction neighborhood size on regular lattices [5].

We have not attempted to optimize the relative length of the IP_0_ information gain phase, and it is clear in some contexts that finer-tuned play is possible, particularly against generous ZD strategies for the donation game. In particular, very small populations may require a more refined information gain phase. We have also not attempted to optimize against non-memory-one strategies.

### History of Play

In [20], Stewart and Plotkin argue (under weak selection, in the absence of ambient noise, and using stationary score as fitness) that one need only consider memory-one strategies in population games to determine evolutionary robustness (extending a similar idea of Press and Dyson for two-player games). However, this view appears incomplete both theoretically (c.f. Adami and Hintze’s Conditional Defector [13], or our analysis of the advantages of frequency-dependent strategy optimization), and empirically (e.g. our IP_0_ simulation data). Our results suggest that in population games it is not generally sufficient to consider only memory-one players—unless non-memory-one strategies are axiomatically forbidden, e.g. by asserting that no player can track its history of game outcomes versus another player.

Another important distinction of IP_0_ is that the individual information players cannot be aggregated as all having the same stationary score with each other. Indeed, the IP_0_ subpopulation is more like a quasispecies with several closely related variants, with each information player potentially identifying a different subpopulation of information players and having inferred slightly different conditional probability vectors for non-IP_0_ strategies (the information players share no information). Accordingly, we computed fixation probabilities empirically from large numbers of simulations and cannot rely on the typical analytic formulas for two-type population games (death-birth processes). For larger populations, the deviation from the theoretical values (from the stationary payouts) should be small, since IP_0_ can quickly approach stationary payoffs.

We believe it will be interesting to explore the space of possible information player strategies. For example, against higher-order Markov strategies, such as Tit-for-Two-Tats, several considerations apply. First, IP_0_’s infogain phase can in general recognize such higher-order strategies as being of opposing (non-IP_0_) type as easily as it can for memory-one strategy opponents. Second, an information player can deploy against such strategies a strategy such as ZD or TFT with a long-term performance guarantee (that holds regardless of what Markov order its opponent uses) [8]. Third, an information player could use *potential information* metrics to detect violations of its default memory-one model [22]. As another example, Fischer et al.’s MaRS (mimicry and relative similarity) strategy [23] appears to fit our basic definition of an information player. That is, MaRS uses a unique identifier for each opponent, and records the history of that opponent’s play to formulate a counter-strategy. Otherwise MaRS is quite different from IP_0_. Both MaRS and IP_0_ are likely to exhibit interesting behaviors for asymmetric and higher-dimensional games as the space of strategies beyond memory-one strategies is explored. These questions, and the issue of effective counter-strategies to IP_0_ and other information players, suggest directions for future work.

## Materials and Methods

### Simulations

Evolutionary simulations were performed using either the Moran process or the imitation dynamic with selection strength *σ* = 1 as in [9] [20]. (All reported results used the imitation process, results for the Moran process were similar.) Unless otherwise stated, all simulations were performed with a total population size of *N* = 100 starting with a single player of the invading type and run until fixation of either the resident or invading type, and the donation game score matrix (2, -1, 3, 0) as in [9].

In most cases, *N*_*sim*_ = 10,000 independent simulations were run for each (invader, resident) pair, and p-values for the observed number of successful invasions *k* were computed under a null hypothesis *H*_0_ assuming a neutral rate of fixation *θ* = 1/*N*:

$$\begin{array}{c} p_{>}=p\left(K\geq k|H_{0},\theta =1/N\right)=\sum_{K=k}^{N_{sim}}\left(\begin{array}{c} N_{sim}\\ K \end{array}\right)\theta^{K}{\left(1-\theta \right)}^{N_{sim}-K} \end{array}$$

Following [8] and [9], we focus on memory-one strategies with probability vector

$$\begin{array}{c} p=\left(p_{1},p_{2},p_{3},p_{4}\right)=\left(Pr\left(C|CC\right),Pr\left(C|CD\right),Pr\left(C|DC\right),Pr\left(C|DD\right)\right). \end{array}$$

Unless otherwise specified in the text, we used the standard probability vector specified in [9] for ZDR (with $\kappa =2,\chi =\frac{1}{2},\phi =0.1$), and ZD_*χ*_ (with $\kappa =0,\chi =\frac{1}{2},\phi =0.1$).

### Information Player Implementation

We implemented a basic information player strategy, called IP_0_, with the following components: (1) an *infogain* phase during which an IP_0_ player chooses its moves to maximize its information yield about a new player, both to assess whether it is another IP_0_ (self vs. non-self), and in the latter case to estimate its strategy vector; (2) a *groupmax* phase during which IP_0_ seeks to maximize its score relative to the opponent group, by either cooperating (if the other player is also IP_0_) or using its current optimal strategy versus the group (if the other player is not an IP_0_). Note that when multiple IP_0_ players are present in a population, they operate completely independently; they do not share information or communicate.

### Basic definitions

IP_0_ records the outcomes of its games vs. a given player in terms of (*n*_*AB*_, *m*_*AB*_) pairs, where *A* is a possible move (C or D) by itself, *B* is a possible move by the other player (C or D), *n*_*AB*_ is the total number of times game outcome *AB* has occurred with this player, and *m*_*AB*_ is the number of those cases where the other player’s next move was C. Treating each such case as a binomial event with probability *p*_*AB*_ = *θ* (probability of cooperating given game outcome AB), the posterior distribution is *p*(*θ*∣*n*_*AB*_, *m*_*AB*_) = *β*(*m*_*AB*_ + 1, *n*_*AB*_ − *m*_*AB*_ + 1) (i.e. the Beta distribution assuming a uniform prior *p*(*θ*) = 1), the maximum likelihood estimator is *θ̂* = *m*_*AB*_/*n*_*AB*_, and the posterior expectation value is $\bar{\theta }=E(\theta |n_{AB},m_{AB})=(m_{AB}+1)/(n_{AB}+2)$. We use the symbol $\bar{p}=(\bar{\theta_{CC}},\bar{\theta_{CD}},\bar{\theta_{DC}},\bar{\theta_{DD}})$ to refer to such an infered probability vector.

### Infogain phase

For the first 10 rounds of its play with another player, IP_0_ chooses its moves to seek game outcomes *AB* about which it has the *least* information (smallest number of counts *n*_*AB*_). Specifically, if the current game outcome was *ab*, then it chooses the move *A* that minimizes the expectation value of *n*_*AB*_:

$$\begin{array}{c} A_{infogain}=\arg\min_{A}E\left(n_{AB}|ab\right)=\arg\min_{A}\sum_{B}p\left(B|ab\right)n_{AB} \end{array}$$

where *p*(*B*∣*ab*) = (*m*_*ab*_ + 1)/(*n*_*ab*_ + 2) for *B* = C. In the case of exact ties (equal expectation values for *A* = C,D), the IP_0_ breaks the tie by computing the MD5 hash value of the game outcomes history string (e.g. CCDC…), and choosing C if its least-significant bit is zero, otherwise D. Note that since this rule depends only on information known to both players (their game outcomes), IP_0_ can predict what moves the other player would choose if it too were an IP_0_. (Of course, in the presence of noise *ε*, the confidence of this prediction drops to 1 − *ε*).

### Groupmax phase

During this phase, IP_0_ seeks to maximize its average relative score vs. the opposing group *G* (Equation 1). Each IP_0_ seeks to maximize *S*_*II*_ (average score versus other IP_0_ players by cooperating with any player it believes to be an IP. With all other players, it applies its current groupmax probability vector *p*_*groupmax*_ chosen to maximize the difference between the second (*S*_*IG*_) and third (*S*_*GI*_) terms above (see below for details).

### Tag inference

After each game, IP_0_ computes the likelihood odds ratio for the observed move *B* of the other player assuming either that it is also an IP_0_, or that is a member of the opponent group (GP). This is used to update the total log-odds ratio for that player:

$$\begin{array}{c} L\prime =L+\log\frac{p(B|IP_{0},\epsilon )}{p(B|\text{GP})} \end{array}$$

where *L* is the current log-odds ratio, *L*′ is the new log-odds ratio, and *ε* is the error rate (frequency at which a player’s moves are flipped).

During infogain phase, the move expected from an IP_0_ player is predicted by the infogain model. During groupmax phase, it is predicted by a Hidden Markov Model (HMM) [24] consisting of just two states: ALLC (“the other IP_0_ recognizes me as an IP_0_, and hence cooperates with me”); and *p*_*groupmax*_ (“the other IP_0_ believes I am not IP_0_, and hence applies *p*_*groupmax*_ against me”). The HMM permits a transition between either of these states with 1% probability per round. At the beginning of groupmax phase, the prior probability of the ALLC state is simply set to the current posterior probability that the other player will classify me as an IP, specifically *p*(ALLC) = 1/(*e* ^−*L*^+1), where *L* is the log-odds ratio the other player would compute from my moves.

The conditional probability *p*(*B*∣GP) is computed according to $\bar{p}$, the current inferred strategy of the opponent. If IP_0_ has not yet confidently identified any players as GP (see below for details), then this $\bar{p}$ is derived solely from the IP player’s game outcomes with this specific player. Otherwise, $\bar{p}$ is computed from game outcomes vs. all GP players that it has confidently identified. This assumes that all non-IP_0_ opponents use the same strategy and could be relaxed for games with more than two types.

During infogain phase, an IP_0_ player classifies another player as confidently GP, based on the p-value of its history of moves under the null hypothesis that it is an IP_0_ playing infogain moves:

$$\begin{array}{c} p\left(E\geq e|n,\epsilon \right)=\sum_{E=e}^{n}\left(\begin{array}{c} n\\ E \end{array}\right)\epsilon^{E}{\left(1-\epsilon \right)}^{n-E}\leq \alpha \end{array}$$

where *n* is the number of games it has played vs. that player, *e* is the number of observed mismatches vs. the expected infogain move (during those games), *E* is the associated random variable, and *ε* is the error rate. We used *α* = 0.01, for at most one expected false positive (in a population of at most 100 IP_0_). During groupmax phase, an IP player classifies each player according to its current log-odds ratio: as an IP_0_ if *L* > 0, otherwise as a GP. Finally, it estimates the total number of IPs currently in the population from its posterior expectation value:

$$\begin{array}{c} \bar{m}=1+\sum_{i}p(IP_{0}|L_{i})=1+\sum_{i}\frac{1}{e^{-L_{i}}+1} \end{array}$$

where *L*_*i*_ is its log-odds ratio for the hypothesis that player *i* is IP_0_ vs. is a GP (the one additional count is for the IP_0_ player itself). When the IP detects birth of a new player, it initializes the new player’s prior log-odds ratio to $L=\log\frac{\bar{m}}{N}$. When it detects the death of a player, if it was confidently a GP (*L* < log *α*), that player’s outcome counts (*n*_*AB*_, *m*_*AB*_) are saved for inclusion in future computations of the GP strategy vector $\bar{p}$.

### Groupmax strategy optimization

If an IP_0_ is in groupmax phase with at least one player, it computes an optimal strategy to use against the opposing group, based on its estimate of the total number of IP_0_ ($\bar{m}$) and its estimate of the opponent group’s strategy vector ($\bar{p}$). It does this based on seeking the strategy *p*_*groupmax*_ that maximizes the interaction terms of the relative score:

$$\begin{array}{c} p_{groupmax}=\arg\max_{q}\left[\frac{N-\bar{m}}{N-1}S\left(q,\bar{p}\right)-\frac{\bar{m}}{N-1}S\left(\bar{p},q\right)\right] \end{array}$$ (2)

where *S*(*p*, *q*) is the theoretical long-term score for strategy vector *p* when playing against strategy vector *q*. We compute *S*(*p*, *q*) as previously described by [8]. Briefly, a game between any two players is a Markov chain with states as pairs of plays in each round {*CC*, *CD*, *DC*, *DD*}. The chain has a unique stationary distribution **s**, and the mean of any four-vector *f* = (*f*_1_, *f*_2_, *f*_3_, *f*_4_) with the stationary distribution for two players *p* and *q* is given by the Press and Dyson determinant [8]

$$\begin{array}{c} D\left(p,q,f\right)=\text{det}\left[\begin{array}{cccc} -1+p_{1}q_{1} & -1+p_{1} & -1+q_{1} & f_{1}\\ p_{2}q_{3} & -1+p_{2} & q_{3} & f_{2}\\ p_{3}q_{2} & p_{3} & -1+q_{2} & f_{3}\\ p_{4}q_{4} & p_{4} & q_{4} & f_{4} \end{array}\right] \end{array}$$ (3)

when *f* gives the scores that player *p* would receive for outcomes (CC, CD, DC, DD) respectively. Using this expression, IP_0_ simply searches the 4-dimensional strategy vector space by gradient descent for the *p* that maximizes the relative score vs. the opponent strategy $\bar{p}$.

Our implementation of IP_0_ and the simulation code used for this manuscript is available at https://github.com/cjlee112/latude.
